# Alternative strategy for TAVR in the context of limited peripheral vascular access: a case report

**DOI:** 10.3389/fcvm.2025.1546342

**Published:** 2025-05-22

**Authors:** Liang Liu, Yi-Jing Guo, Xian Jin

**Affiliations:** Department of Cardiology, Shanghai Sixth People’s Hospital Affiliated to Shanghai Jiao Tong University School of Medicine, Shanghai, China

**Keywords:** TAVR, vascular route, artificial vascular, antegrade approach, retrograde approach

## Abstract

**Background:**

Severe aortic valve stenosis (AS) has become one of the leading causes of mortality in elderly patients. Currently, transcatheter aortic valve replacement (TAVR) has emerged as a crucial treatment strategy for patients with severe AS. However, numerous patients face difficulties receiving TAVR procedure due to individual patient characteristics and peripheral vascular conditions.

**Case report:**

We present a case report of an elderly patient with low body weight and low-risk severe AS. In this patient, the peripheral blood vessels, particularly both iliac arteries, exhibited a notably small diameter throughout. Additionally, the aortic valve area was reduced, with valve leaflets severely calcified, significantly compromising their mobility. The patient opted for TAVR procedure. However, the traditional femoral-iliac approach for TAVR was constrained by the patient's peripheral vasculature and low BMI. Consequently, we established an artificial vascular-common iliac artery pathway as the primary access route for completing the procedure. Intraoperatively, the retrograde guidewire failed to traverse the patient's valve. As a result, an antegrade approach was adopted in combination with an *in vitro* guidewire capture technique, and ultimately, the TAVR operation was completed.

**Conclusion:**

Our case demonstrates an innovative interventional treatment strategy for TAVR in patients with severe AS, peripheral vascular limitations, and a low BMI, for whom retrograde wire passage through the valve is not feasible.

## 1 Introduction

Aortic stenosis (AS) ranks among the most prevalent valvular heart diseases. It usually stems from structural anomalies of the aortic valve, which cause the valve orifice to narrow. This narrowing raises the resistance to left ventricular outflow and ultimately triggers pathological remodeling of the left ventricle. Patients with severe AS may experience angina pectoris, syncope, heart failure, and other symptoms, leading to a poor prognosis. The primary treatment strategy for severe AS is artificial valve replacement. In the past, severe AS was treated by open-heart surgery for valve replacement, known as Surgical Aortic Valve Replacement (SAVR). However, with advancements in medical technology and device innovation, Transcatheter Aortic Valve Replacement (TAVR) has emerged as one of the treatment options for severe AS, particularly for patients with intermediate to high risk (1). Compared to SAVR, TAVR offers advantages such as minimally invasive, safer, faster recovery, and with fewer complications (1). Nevertheless, in clinical practice, many patients with severe AS face challenges in TAVR procedure due to limitations in peripheral vascular conditions. This case report presents a new approach to TAVR for patients with severe AS who have limited peripheral vascular conditions.

## 2 Case description

### 2.1 General information

A 71-year-old female patient was admitted to our hospital due to “repeated syncope for 2 years”. Over the past two years, the patient's primary symptom was syncope accompanied by loss of consciousness, occurring without apparent precipitating factors. There were no symptoms of chest pain or palpitations, and consciousness was regained after a few seconds. The symptoms recurred frequently. Echocardiography in the external hospital revealed senile calcification of the aortic valve with severe stenosis. Consequently, the patient was referred to our department for further management. The patient denied any history of hypertension, diabetes, coronary heart disease, heart failure, and so forth. She also had no history of smoking or alcohol consumption. The patient's height was 165 cm and weight was 35 kg, resulting in a BMI of 12.86. Upon examination, the patient was alert and oriented, with stable respiration and moist skin. Her pulse rate was 76 beats per minute, respiratory rate was 20 breaths per minute, and blood pressure was 88/64 mmHg. Lung auscultation revealed no obvious rales. The heart rhythm was regular, with P2 equal to A2. A grade 4 ejection systole murmur was audible in the aortic valve auscultation area. The abdomen was soft and non-tender, with no rebound tenderness. Mild edema was present in both lower extremities.

### 2.2 Examination and treatment strategy

**Figure 1:**
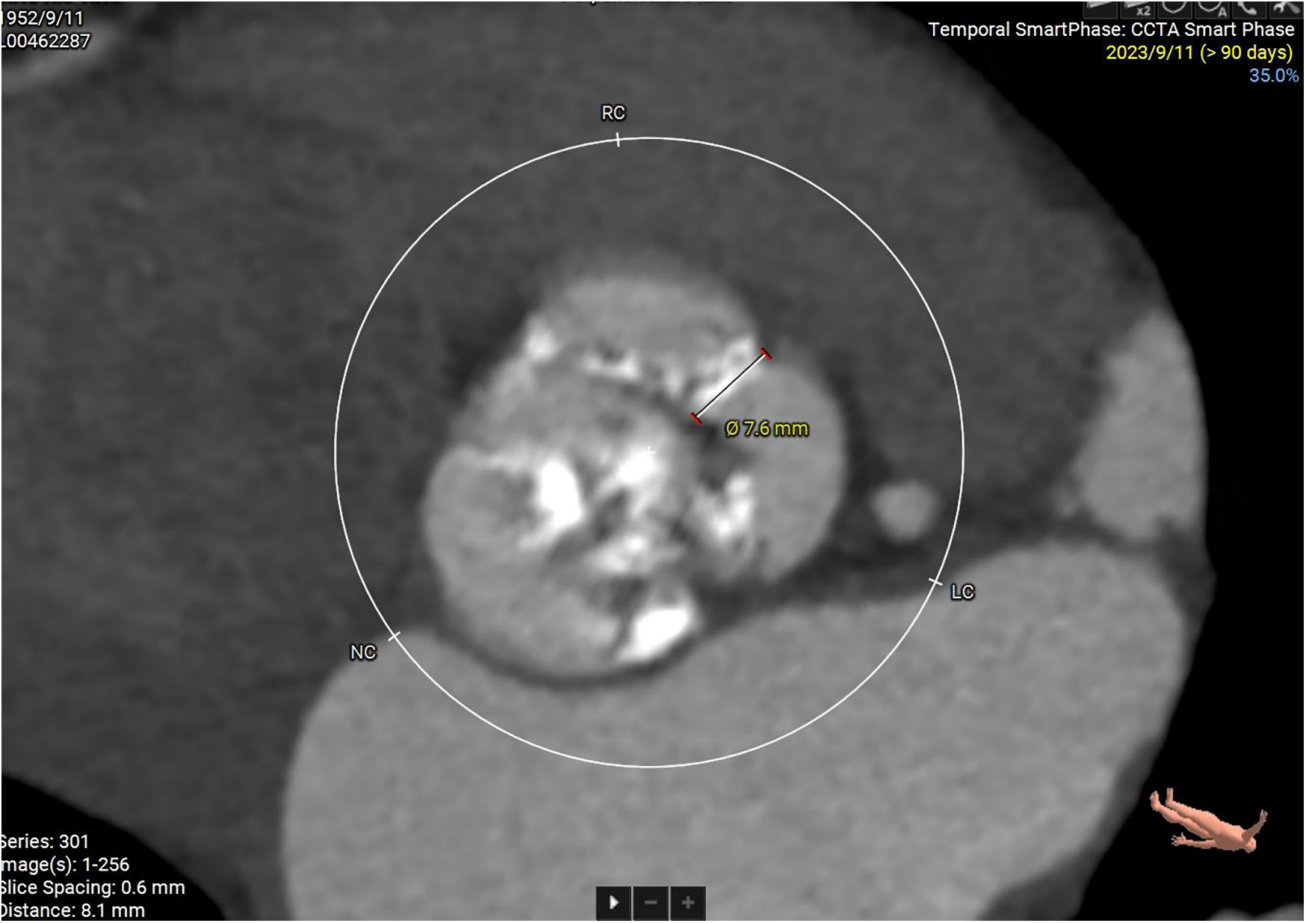
Enhanced CT examination of valve characteristics.

**Figure 2:**
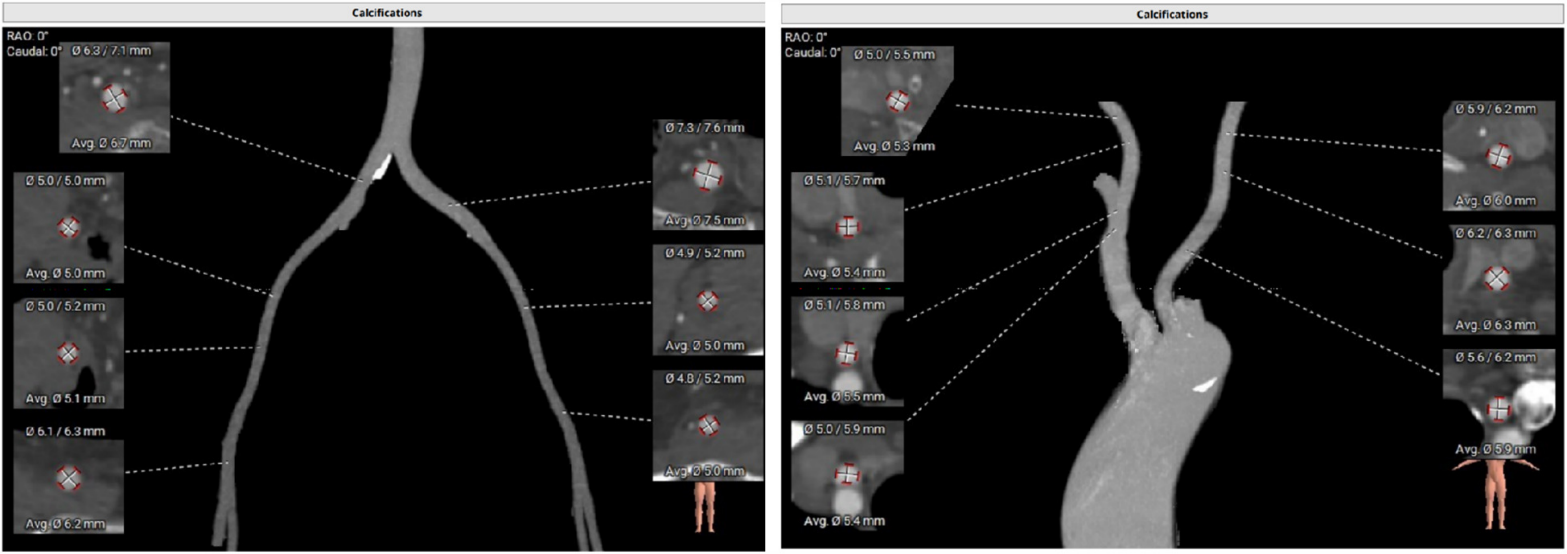
Enhanced CT examination of both common iliac arteries and carotid arteries.

Some important biochemical test results of this patient were presented in the Table 1. Other biochemical indicators, including liver function, electrolytes, coagulation function, and glycated hemoglobin were all within the normal range. The electrocardiogram revealed that she had a sinus rhythm, with ST segment depression ranging from 0.05 to 0.10 mV in leads V3–V6, accompanied by T wave abnormalities. Our hospital's echocardiography results were also listed in the Table 1. The echocardiography conclusion indicated severe AS accompanied by mild regurgitation and the aortic valve was bicuspid aortic valve. The pre-TAVR enhanced CT scan demonstrated that the patient had a Type-1 bicuspid aortic valve with L-R fusion, and the thickness of the fusion raphe was 7.6 mm (Figure 1). All other important data of enhanced CT were listed in the Table 1. The calcification score of the valve leaflets was 723 mm^3^ and the valve orifice area was small, indicating poor leaflet coaptation, compromising their mobility and a high risk of paravalvular leak (Supplementary Video S1). Notably, both common iliac arteries were congenitally thin along their entire lengths. The thinnest part on both sides measured approximately 5.0 mm, and no atherosclerosis was observed (Figure 2). The remaining portions of the abdominal aorta and ascending aorta were normal. The minimum internal diameter of the right carotid artery was 5.0 mm, and that of the left was 5.6 mm (Figure 2). Taken together, the patient's diagnosis was severe AS with mild regurgitation, and the cardiac function was classified as class I. After a thorough evaluation, valve replacement was needed for this patient. The patient and their family opted for TAVR instead of SAVR. However, the TAVR procedure presented certain challenges. The peripheral vascular access was extremely narrow, potentially making it unsuitable as a route for the large TAVR sheath. Furthermore, the aortic valve showed severe calcification and had a small valve area, significantly limiting their mobility. This indicated a considerable difficulty in the retrograde crossing of the valve through the aorta with a guidewire. Our TAVR procedure comprised two key steps: firstly, establishing an artificial vascular-common iliac artery approach, and secondly, proceeding with anterograde atrial septal puncture if the retrograde transaortic approach was unsuccessful.

**Table 1:** Partial biochemical data, echocardiogram data, and CT data.

Variables	Value
Hemoglobin	115 g/L
Platelet	186 × 10^−^9/L
c-TnI	0.01 μg/L
CK-MB	0.9 μg/L
MYO	18.7 μg/L
Creatinine	54 μmol/L
NT-proBNP	2,970 ng/L
Lp(a)	19.1 mg/dl
AVA	0.52 cm^2^
Vmax	6.28 m/s
Vmean	4.3 m/s
PGmax	158 mmHg
PGmean	86 mmHg
LVEF	70%
Aortic annulus diameter	24 mm
RCA height	12.4 mm
LCA height	11.1 mm
STJ diameter	27.9 mm
Valsalva sinus diameter	31.9 mm
Cardiac horizontal angle	48°
Ascending aorta diameter	38.9 mm
Calcium score	723 mm^3^

AVA, aortic valve annulus; V, velocity; PG, pressure gradient; LVEF, left ventricular ejection fraction; RCA, right coronary artery; LCA, left coronary artery.

### 2.3 TAVR procedure

**Figure 3:**
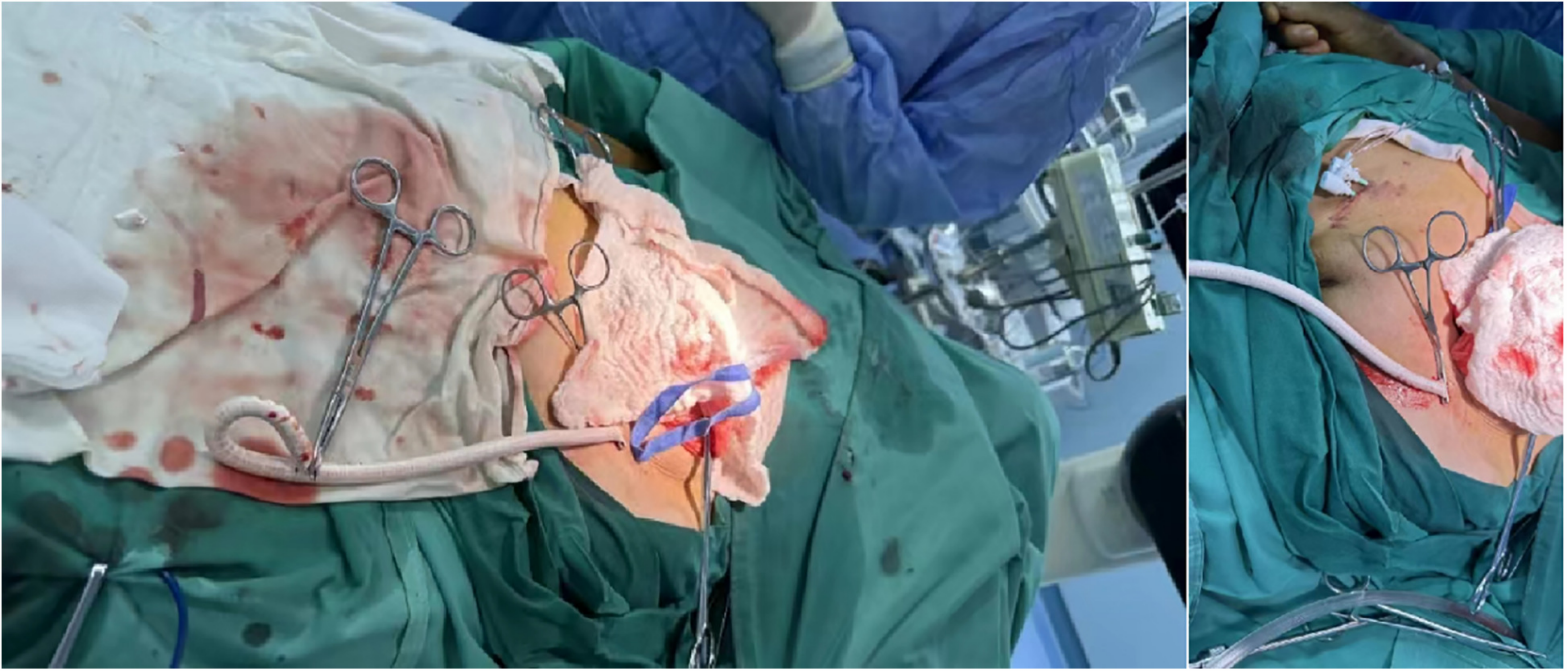
Artificial vessel—common iliac artery TAVR main route.

**Figure 4:**
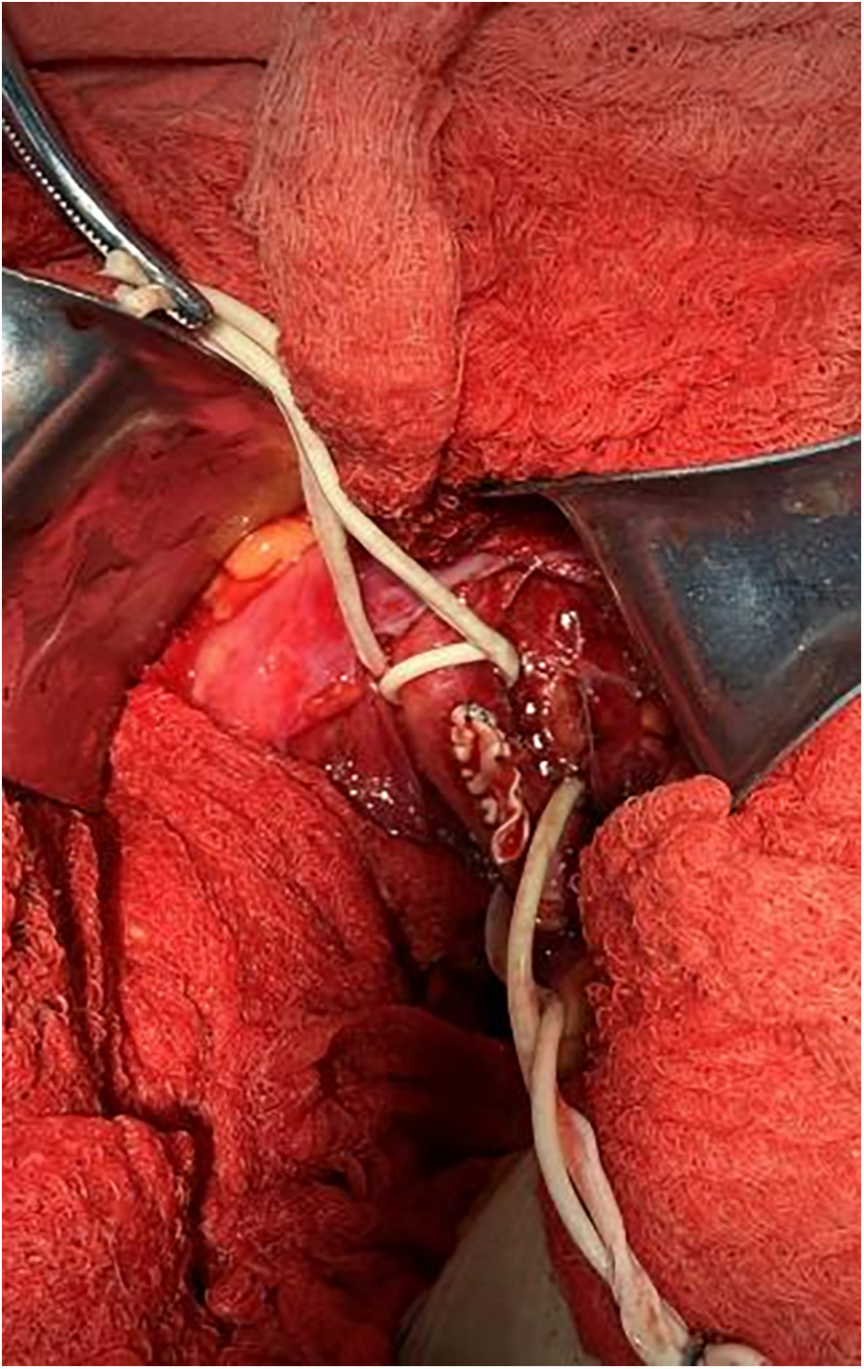
Depicts the suturing of artificial blood vessels with the incision subsequently closed.

1. After the patient had received adequate peripheral intravenous fluid one day prior, bilateral common iliac arteriography and QCA measurement revealed that the narrowest diameters of the left and right common iliac arteries were 5.00 mm and 5.03 mm, respectively (Supplementary Videos S2, S3).
2. Based on preoperative CT examination for TAVR, the Vitaflow® Transcatheter Aortic Valve Replacement system was selected. Considering that the ratio of the 20F sheath to the peripheral blood vessels, including the common iliac artery, was large, and the patient was a thin female, the risk of vascular complications was significantly increased. Therefore, the artificial vascular-common iliac artery approach was performed. The left rectus muscle was incised in the middle. The proximal part of the left common iliac artery was anastomosed with the end of an 8-mm artificial blood vessel. Subsequently, an incision was made in the left lower abdomen to expose the artificial blood vessel, which served as the main access route for the TAVR procedure (Figure 3).
3. The artificial vessel-common iliac artery was used as the main route to send the pigtail catheter to the ascending aorta. A super-stiff guidewire was exchanged, and a 20F large sheath was advanced along it. Attempts to cross the valve using AL1.0, AL0.75, JR3.5, and MPA2 catheters with straight loach guidewire were unsuccessful, prompting consideration of a retrograde wire snaring technique. The interatrial septum was punctured to send the JR3.5 catheter into the left ventricle (Supplementary Video S4). A 260 cm loach guide wire was sent to the descending aorta through the JR catheter and then captured and externalized by the snare through the 20F large sheath (Supplementary Video S5). Afterward, the pigtail catheter was advanced along the loach guide wire into the left ventricle, and subsequently, the TAVR procedure was completed. Then the artificial vessel was trimmed, and the incision was sutured (Figure 4). The procedure was successful and the patient was safely returned to the ward.

### 2.4 Treatment outcomes, follow-up, and prognosis

During the postoperative hospitalization, the patient was monitored by ECG and blood pressure and received symptomatic treatment. The patient did not experience recurrent syncope or other symptoms such as chest pain or tightness. Ward echocardiography demonstrated mild regurgitation of the artificial aortic valve. The average aortic valve pressure gradient was 7 mmHg and the mean velocity was 1.23 m/s. No bleeding or leakage was observed at the abdominal incision site. At discharge, NT-proBNP levels decreased to 957 ng/L, and other laboratory tests were normal. Holter monitoring revealed an average heart rate of 68 bpm, with no atrioventricular block or dynamic ST-T change. Three months later, during a follow-up visit to our clinic, the patient reported no recurrence of syncope or other symptoms since discharge. NT-proBNP was 673 ng/L, and no abnormalities were found in the blood routine and liver and kidney function.

## 3 Discussion

In this case, the diagnosis of severe AS was definitive, and the STS score was 2.35%. This indicated that the patient was a low-risk candidate for surgery. According to current guidelines, TAVR procedure has emerged as the preferred treatment strategy for middle and high-risk severe AS patients (1, 2). However, the use of TAVR in low-risk severe AS patients remains controversial. The results of PARTNER 3 and Evolut Low-Risk Trials demonstrated that, in low-risk severe AS patients, TAVR had outcomes comparable to those of SAVR (3, 4). Given that the patient and their family refused SAVR and opted for TAVR, after a comprehensive evaluation by the heart center, the decision was made to proceed with the TAVR treatment strategy.

In addition to patient selection, the choice of the TAVR access route is also crucial for the success of the operation. The primary route for TAVR procedure is the femoral-iliac artery. A recent review, which encompassed data from 23 original studies of patients who underwent TAVR procedure between 2006 and 2020, revealed a significant increase in peripheral vascular complications when the sheath-to-iliofemoral artery ratio exceeded 0.91 to 1.19 (5). In this case, the iliac arteries were small throughout, with the narrowest portion measuring only 5.0 mm in diameter. Based on our preoperative TAVR evaluation and the selected valve, the ratio of the required sheath-to-iliofemoral artery was notably greater than 1.19. Furthermore, previous studies have shown that patients with low BMI have a high incidence of peripheral vascular complications following TAVR procedure. In this case, the patient's BMI was only 12.86. Although in some centers, TAVR procedure is carried out even when the directly measured diameter of the femoral-iliac artery is less than 5 mm, any incidence of vascular complications can be life-threatening. Nowadays, with the advancement of medicine, commercially available products with a lower external profile, such as the CoreValve™ and Evolut™ TAVR Systems, are available, which also allows for sheathless navigation in 5 mm anatomies. For this case, because the iliac artery has no obvious atherosclerosis and calcification, the above valve systems can be attempted to be selected. However, for this case, we need to comprehensively consider multiple factors, including the price of the artificial valve, the ease of obtaining it (The above TAVR valve was not on the hospital's procurement list at that time, and it could not be normally purchased and used.), the situation with suppliers, and the patient's financial capacity, among others. Therefore, after thorough consideration, we ultimately chose the Vitaflow® Transcatheter Aortic Valve Replacement System for this patient. Together, utilizing the femoral-iliac artery as the TAVR access route posed a higher risk of peripheral vascular complication for this patient, necessitating the exploration of an alternative access route.

When the femoral-iliac artery is not viable, the carotid artery, subclavian artery, and axillary artery can be considered as alternatives (6). However, the preoperative enhanced CT results indicated that the minimum diameters of the carotid and subclavian arteries of this patient were approximately 5 mm, making them unsuitable as the surgical access when we choose the Vitaflow® Transcatheter Aortic Valve Replacement System. A report has described TAVR procedure performed through an intubation of the ascending aorta following a small thoracic incision (7). However, due to the unpredictable consequences of vascular complications in the ascending aorta, this route maybe unsuitable. Another recent case report published in the Chinese Journal of Cardiology pointed out that in a severe AS patient with femoral artery stenosis and severe calcification, the shockwave balloon was used to pretreat the femoral artery. Subsequently, the femoral artery was utilized as an access route to complete the TAVR procedure. However, this method cannot be used in our case. The patient's peripheral arteries are congenitally small, rather than severely stenosed due to calcified plaques. In addition, forcibly expanding the femoral-iliac artery with a shockwave balloon may cause vascular dissection and lead to vascular lesions. In our case, in collaboration with the heart center and vascular surgery department, an artificial vascular-common iliac artery (8 mm) was made as the primary access route for completing the TAVR. The advantages of this method include: (1) minimal surgical trauma and rapid postoperative recovery; (2) no involvement of the aorta, ensuring high safety, and facilitating easier hemostasis in the event of vascular complications. This case presents a novel therapeutic approach for severe AS patients with a high risk of TAVR-related peripheral vascular complications as the primary access route.

The mainstream strategy for TAVR is typically the reverse approach, involving a guidewire crossing the aortic valve via the femoral artery route to facilitate the insertion of the artificial valve. However, in this patient, despite successfully establishing the retrograde access and with the support of various catheters, the loach guidewire could not cross the valve. This was because the valve leaflets were severely calcified and the valve orifice area was small. Therefore, it was more difficult for the guidewire to cross the valve. In addition, because the artificial vascular bypass technique was selected, it was worried that there will be more bleeding after heparinization. Therefore, the time for crossing the valve could not be too long to avoid excessive bleeding. After a thorough analysis of the patient's condition, the team adopted the anterograde transvalvular technique. Finally, the TAVR procedure was completed.

In summary, for patients with severe AS who are undergoing TAVR procedure, preoperative enhanced CT evaluation is of crucial importance, and the selection of the access route is of vital significance. In case of intraoperative complications, it is essential to identify suitable alternative strategies calmly. Furthermore, close teamwork is also essential for the success of TAVR. This case demonstrates a novel treatment strategy for patients who encounter difficulties with peripheral vascular access during TAVR procedure and are unable to cross the valve via the retrograde approach. It offers a potential solution for the clinical management of more patients with severe AS.

## Data Availability

The raw data supporting the conclusions of this article will be made available by the authors, without undue reservation.
