# Supplementary figures and images for: Alternative strategy for TAVR in the context of limited peripheral vascular access: a case report
Source: Front Cardiovasc Med. 2025 May 22;12:1546342. doi: 10.3389/fcvm.2025.1546342 (PMC12137235; doi:10.3389/fcvm.2025.1546342)

## Slide 1
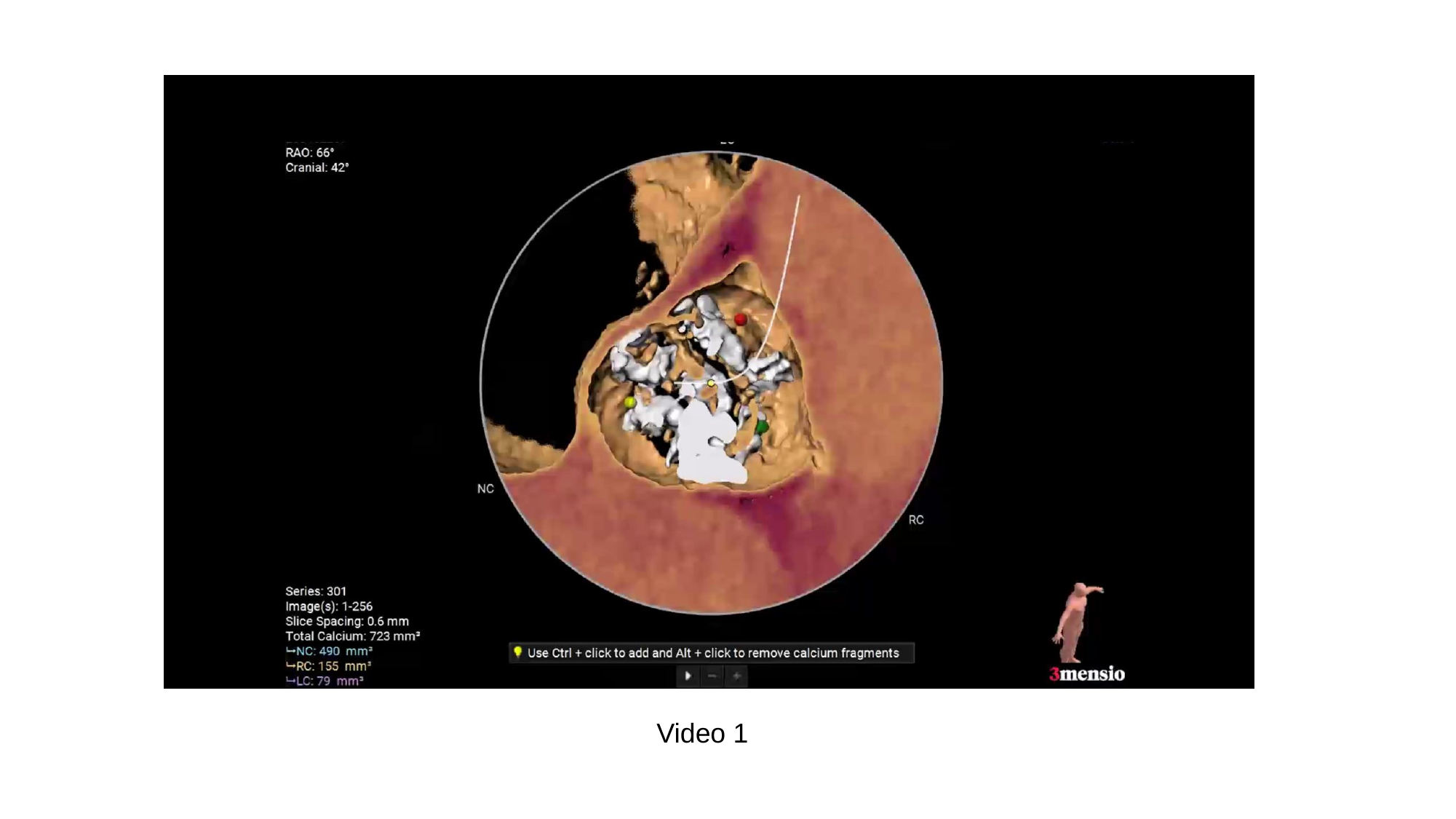

Video 1

## Slide 2
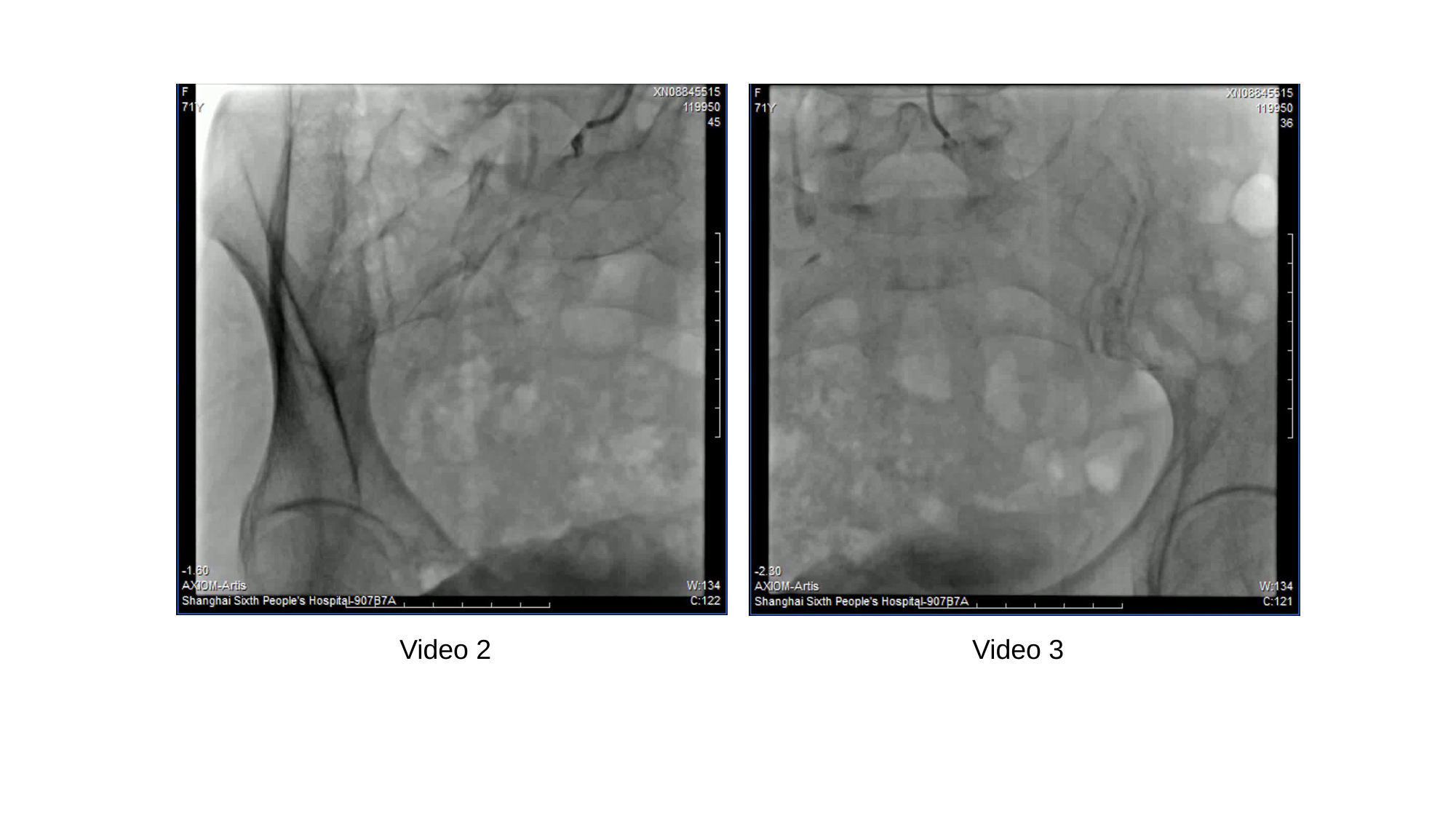

Video 2
Video 3

## Slide 3
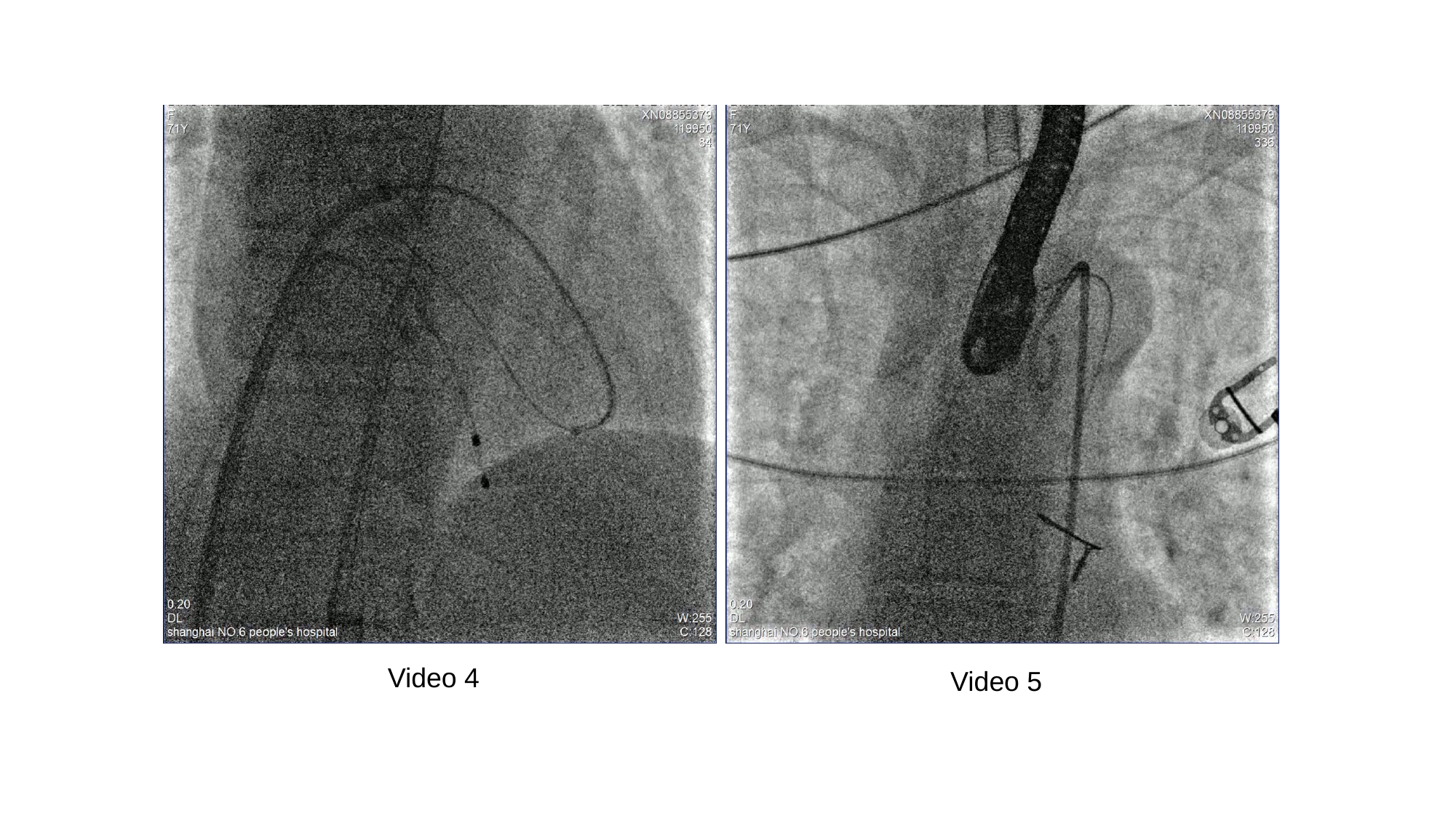

Video 4
Video 5

Supplement: Supplementary file 1 [file Presentation1.pptx]
